# The Immunological Role of CDK4/6 and Potential Mechanism Exploration in Ovarian Cancer

**DOI:** 10.3389/fimmu.2021.799171

**Published:** 2022-01-14

**Authors:** Chen Liu, Yuhan Huang, Yaoyuan Cui, Jun Zhou, Xu Qin, Li Zhang, Xi Li, Yuan Li, Ensong Guo, Bin Yang, Xi Li, Junpeng Fan, Xiong Li, Yu Fu, Si Liu, Dianxing Hu, Rourou Xiao, Zizhuo Wang, Yingyu Dou, Wei Wang, Wenting Li, Xiaohang Yang, Jingbo Liu, Wenju Peng, Tianyu Qin, Lixin You, Funian Lu, Chaoyang Sun

**Affiliations:** ^1^ Department of Obstetrics and Gynecology, Tongji Hospital, Tongji Medical College, Huazhong University of Science and Technology, Wuhan, China; ^2^ Department of Obstetrics and Gynecology, Shanghai General Hospital, Shanghai Jiao Tong University School of Medicine, Shanghai, China; ^3^ Department of Pediatrics, Tongji Hospital, Tongji Medical College, Huazhong University of Science and Technology, Wuhan, China

**Keywords:** CDK4/6, ovarian cancer, immune, ncRNAs, STING

## Abstract

**Background:**

Ovarian cancer (OC) is one of the most lethal gynecologic cancers. Growing evidence has proven that CDK4/6 plays a key role in tumor immunity and the prognosis of many cancers. However, the expression and function of CDK4/6 in OC remain unclear. Therefore, we aimed to explore the influence of CDK4/6 in OC, especially on immunity.

**Methods:**

We analyzed CDK4/6 expression and prognosis using The Cancer Genome Atlas (TCGA), Gene Expression Omnibus (GEO) and Genotype Tissue Expression (GTEx) data. Subsequently, we used the cytoHubba plug-in of Cytoscape software and starBase to identify the noncoding RNAs (ncRNAs) regulating CDK4/6. Finally, we verified the effect of CDK4/6 on immunity in OC cell lines and animal models.

**Results:**

CDK4/6 expression was higher in OC tissues than in normal ovarian tissues, and the high expression levels of CDK4/6 contributed to the immunosuppressive state of OC and were thus related to the poor prognosis of OC patients. This was also in general agreement with the results of OC cell line and animal experiments. Mechanistically, the CDK4/6 inhibitor palbociclib increased the secretion of interferon (IFN)-γ and the interferon-stimulated gene (ISG) response, thereby upregulating the expression of antigen-presenting molecules; this effect was partly dependent on the STING pathway and thus activated immunity in OC. Additionally, according to public data, the LRRC75A-AS1-hsa-miR-330-5p axis could inhibit the immune response of OC patients by upregulating CDK4/6, leading to a poor prognosis.

**Conclusion:**

CDK4/6 affects the immune microenvironment of OC and correlates with the prognosis of OC patients.

## Introduction

Ovarian cancer (OC), famous for its poor prognosis, is considered a “silent killer” since most cases are diagnosed in an advanced stage (1, 2). Many risk factors related to the prognosis and progression of OC, such as family history, immune system, and oncogene mutations, have been revealed (3). Despite the major improvement in therapy, the overall survival (OS) of patients with OC remains unsatisfactory, with more than 230,000 new cases and 150,000 related deaths of OC every year (4). Therefore, effective therapeutic targets or promising prognostic biomarkers are urgently needed to improve the prognosis of OC patients (5).

CDK4/6 are core cell cycle machinery that drive cell proliferation and are necessary for the initiation and progression of many malignancies (6–8). According to recent reports, CDK4/6 inhibitors could not only induce tumor cell cycle arrest but could also promote antitumor immunity (9–11). A recent report also validated that CDK4/6 inhibitors markedly suppressed the proliferation of regulatory T cells (Tregs) and promoted cytotoxic T cell-mediated clearance of tumor cells in breast cancer (12). In lung cancer, Deng et al. found that CDK4/6 inhibitors increased the tumor-infiltrating level of T cells *in vivo* (13). However, there is no systematic study of the expression levels of CDK4/6 and their implications in the prognosis and progression of OC. Moreover, the associations of CDK4/6 with the tumor immune microenvironment in OC have not been determined.

In this study, we used public data to analyze the expression and prognostic impact of CDK4/6 in many human cancers, including OC. The regulation of CDK4/6 by noncoding RNAs (ncRNAs), namely, microRNAs (miRNAs) and long noncoding RNAs (lncRNAs) were explored in OC. Moreover, we initially explored the mechanism by which palbociclib, a CDK4/6 inhibitor, activated immunity in OC. Taken together, our findings suggest that CDK4/6 affects the immune microenvironment of OC and correlates with the prognosis of OC patients.

## Materials and Methods

### TCGA Data and GEO Data Download, Process, and Analysis

We downloaded the RNA expression and clinical data from the TCGA and GTEx (contains 11,069 samples from 33 types of cancer) database,UCSC Xena (https://xena.ucsc.edu/). We obtained the gene expression profiling data from the GEO database (https://www.ncbi.nlm.nih.gov/gds). The GSE18520 dataset contained the gene expression profiles of 53 OC patients and 10 normal controls. Additionally, we obtained 1,811 unique immune-related genes (IRGs) from the Immunology Database and Analysis Portal (ImmPort) database (https://www.immport.org/home).

### Pathway Enrichment Analysis for the Molecular Function

To elaborate the potential functional annotation and pathway enrichment related to the immune related DEGs, we performed Gene Ontology (GO) analyses, including biological process (BP), cellular component (CC), molecular function (MF), and Kyoto Encyclopedia of Genes and Genomes (KEGG) pathway analyses using the clusterProfiler package in R version 3.6.3 software.

### Candidate miRNA Prediction

Some miRNA target gene prediction programs (PITA, RNA22, miRmap, microT, miRanda, PicTar, and TargetScan) were used to predict upstream binding miRNAs of CDK4/6. Only the predicted miRNAs that targeted CDK4 and CDK6 as mentioned above were used for further analyses. Furthermore, the CytoHubba plug-in was analyzed to screen the significant ncRNAs regulated CDK4/6, and genes with degree >30 were identified as hub ncRNAs in the OC TCGA cohort.

### StarBase Database Analysis

We introduced StarBase (http://starbase.sysu.edu.cn/) to perform expression correlation analysis for miRNA-CDK4/6, which is aimed for exploring ncRNAs-related studies. In this paper, we performed correlation analysis for miRNA-CDK4/6, lncRNA-has-mir330-5p in OC in StarBase.

### TIMER Database Analysis

We used the “Immune-Gene” module of the TIMER2 web server to explore the association between CDK4/6 expression and immune infiltrates across OC. P-value <0.05 was regarded as statistically significant.

### TISIDB Database Analysis

The TISIDB database (http://cis.hku.hk/TISIDB) was aimed for tumor and immune system interaction, which integrated para-cancerous multi-omics data. Here, the TISIDB provided us associations for CDK4/6 with lymphocytes, immunomodulators, and chemokines.

### Kaplan–Meier Plotter Analysis

We used Kaplan–Meier plotter (http://kmplot.com/analysis/) to analyze the prognostic value of the LRRC75A:AS1 in OC patients. Besides, we used CDK4/6 expression data to perform the survival analysis in the R package “survival”. Subsequently, we divided the tumor samples into two groups according to their individual expression level compared with their mean expression level among all tumor samples in the TCGA cohort. In addition, we also explored the correlation between the expression of CDK4 and outcome of the ovarian cancer patient using the PrognoScan database (http://www.abren.net/PrognoScan/).

### Immune Infiltration Analysis by ssGSEA

We performed ssGSEA analysis using R package (version 3.6.3) GSVA to figure out the immune infiltration analysis of OC. Then, we quantified the infiltration levels of 24 immune cell types from gene expression profile in the TCGA OC cohort. Furthermore, to discovery the correlation between ncRNAs-CDK4/6 and the infiltration levels of 24 immune cells, we determined P-values by the Spearman and Wilcoxon rank sum test.

### Cell Culture

OVCAR3 was obtained from the American Type Culture Collection (ATCC), HOC7 was from MDACC characterized Cell line Core, and ID8 was a C57BL/6 mouse-derived cell line of ovarian cancer, given by Professor K. Roby (Department of Anatomy and Cell Biology, University of Kansas, USA). All cell lines were confirmed by short tandem repeat (STR) analysis.

### 2D Cell Viability Assay

A total of 3,000 cells per well were fed on 96-well plates and treated with palbociclib for three days, the cell counting kit 8 (CCK8, Dojindo Laboratories, Japan) was used to detect cell proliferation. The half-maximal inhibitory concentration value (IC50) was calculated by Graphpad8.

### Quantitative RT-PCR (RT-PCR)

We used FastPure^®^ Cell/Tissue Total RNA Isolation Kit (Vazyme) to extract RNA. Then, we used HiScript^®^ Reverse Transcriptase (Vazyme) to reversely transcribe RNA. RT-PCR was performed by cDNA, gene-specific primers and IQ SYRB Green Supermix and detected by iCycler QTX detection system (Bio-Rad). The 2-rCt method was used to quantitate fold changes by normalizing them to Actin.

### ID8 Xenografts

Injected into the peritoneal cavity of 6–8-week-old female C57BL/6J mice were 1 ∗ 10^7^ luciferase ID8 cells (per mouse) in a 1:1 mix of PBS and Matrigel, which were monitored once a week. After palpable tumors formed, drugs were administered daily by vehicle (0.5% hydroxypropyl methylcellulose and 0.2% Tween 80, oral gavage), palbociclib (40 mg/kg, oral gavage) [n = 5 per group]. Mice were treated until Day 28 and sacrificed for tissue harvest. All images were acquired using an Inveon Multi-Modality scanner, a small-animal PET/CT system.

### Immunohistochemistry (IHC) Staining

To evaluate differences in CDK4/6 expression at protein level, on the one hand, IHC images of CDK4/6 in normal ovary and ovarian cancer tissues were downloaded from the HPA (http://www.proteinatlas.org/) and analyzed. On the other hand, we did an immunohistochemistry experiment, and the approaches were as described in the previous study.

### Statistical Analysis

R version 3.6.3 software was used for statistical analyses. Significantly statistical differences were considered when P <0.05 (***P <0.001, **P <0.01, *P <0.05). All gene expression data was normalized by log2 transformation. We used two sets of t-test to compare normal tissue and cancer tissue. The Kaplan–Meier curve was used for survival analyses. We used Spearman’s or Pearson’s test to perform the correlation analysis between the two variables.

## Results

### The mRNA Expression Levels of CDK4/6 in Different Types of Human Cancers

**Figure 1:**
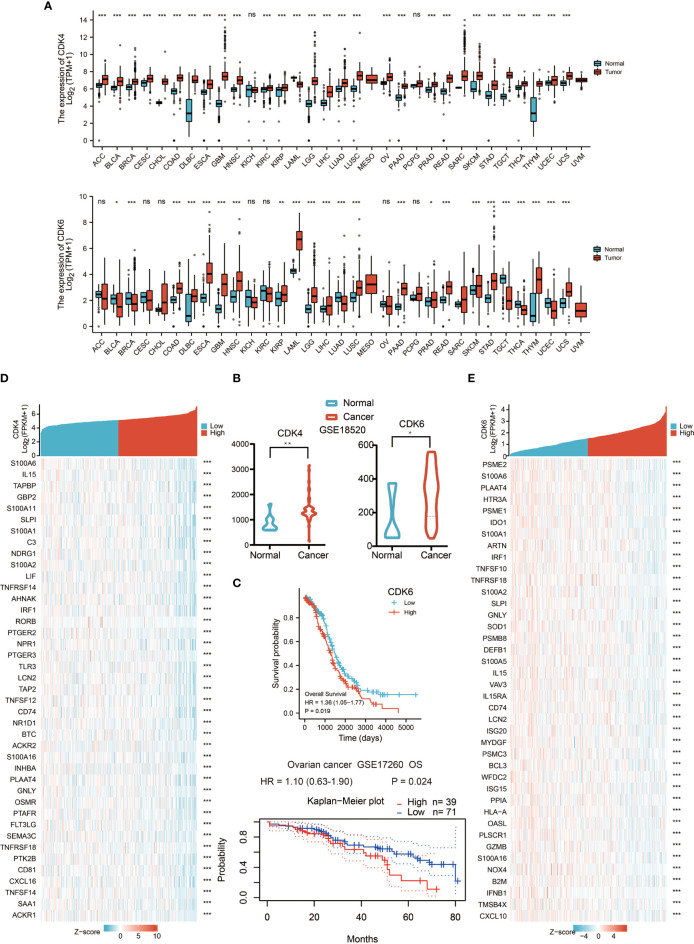
The mRNA expression levels of CDK4/6 in different types of human cancers. **(A)** The expression of CDK4/6 in 33 types of human cancer based on cancer and normal data of the TCGA and GTEx cohorts. *p-value <0.05; **p-value <0.01; ***p-value <0.001; ns, nonsense. **(B)** Comparison of CDK4/6 gene expression between normal and tumor tissues in the GSE18520 dataset. *p-value <0.05; **p-value <0.01; ***p-value <0.001; ns, nonsense. **(C)** Kaplan–Meier analysis of the association between CDK4/6 expression and OS in OC. **(D)** Heat map showing the top 40 genes of CDK4 and immune correlation. **(E)** Heat map showing the top 40 genes of CDK6 and immune correlation.

Initially, we evaluated CDK4/6 transcription levels in different human tumors by analyzing RNA-seq data from The Cancer Genome Atlas (TCGA) and Genotype Tissue Expression (GTEx) databases. Compared with that in normal tissue, CDK4/6 mRNA expression was remarkably higher in tumor tissue overall, which demonstrated that CDK4/6 are abnormally expressed in multiple tumors (**Figure 1A**); however, there was no significant change in some levels between normal tissue and tumor tissue, including CDK6 in OC. We further assessed CDK4/6 expression levels in OC using Gene Expression Omnibus (GEO) data and found that the CDK4/6 mRNA expression levels were significantly higher in OC tissue versus normal tissue (**Figure 1B**). A similar result was demonstrated according to immunohistochemistry (IHC) data obtained from the Human Protein Atlas (HPA) database. CDK4/6 were overexpressed in OC tissues but downregulated in normal ovarian tissues. Since CDK4/6 levels were abnormally higher in OC specimens, we then studied the clinical significance of CDK4/6 expression in OC patients. According to the Kaplan–Meier survival curves, as shown in **Figure 1C**, our data indicated that higher expression of CDK6 was markedly correlated with poor OS in OC patients from the TCGA cohort. Unfortunately, the impact of CDK4 on survival in the TCGA database was not statistically significant. However, the data from GSE17260 suggested that the expression of CDK4 was higher, the prognosis of ovarian cancer patients was poorer (**Figure 1C** **)**.

To explore the influence of CDK4/6 on immunity, we took the intersection of genes negatively related to CDK4/6 and immune-related genes and obtained the top 50 gene sets that were related to CDK4 and CDK6 (**Figures 1D, E** **)**. The results indicated that higher expression of CDK4/6 was related to lower expression of antigen-presenting molecules, chemokines, and other immune components.

### GO and KEGG Enrichment Pathway Analysis

**Figure 2:**
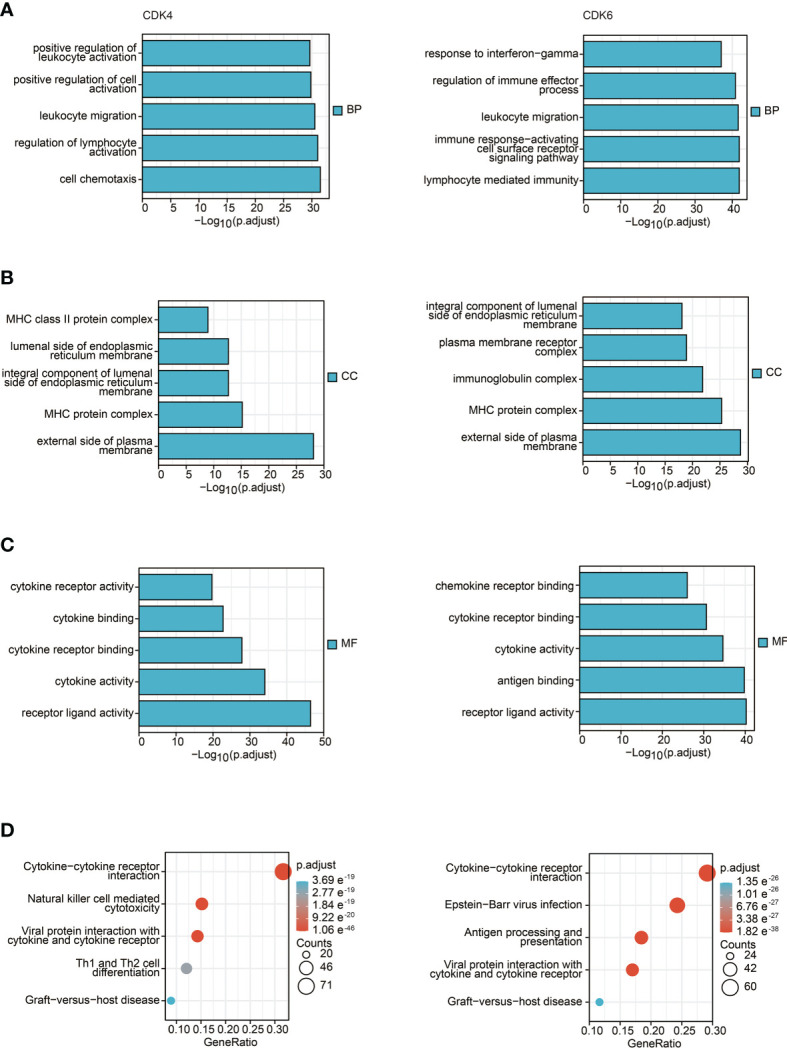
GO and KEGG enrichment pathway analysis. **(A)** Top 5 of biological process enrichment related to CDK4/6 related genes with bar graph. **(B)** Top 5 of cell component enrichment related to CDK4/6 related genes with bar graph. **(C)** Top 5 of molecular function enrichment related to CDK4/6 related genes with bar graph. **(D)** Top 5 of KEGG pathway enrichment related to CDK4/6 related genes with bar graph.

To further explore the influence of CDK4/6 on immunity, we first obtained the top 300 genes that were in the intersection of genes negatively related to CDK4/6 and immune-related genes. Then, we performed KEGG and GO analyses, including biological process (BP), cellular component (CC), and molecular function (MF) analyses. As shown in **Figures 2A–D**, the main enriched BPs were leukocyte migration, lymphocyte-mediated immunity, etc. The MF analysis results suggested that these genes are mainly involved in cytokine activity, cytokine receptor binding, etc. In addition, cellular component (CC) analysis primarily indicated that CDK4/6 were negatively related to the MHC protein complex, external side of plasma membrane, etc. The KEGG pathway analysis showed that genes negatively correlated with CDK4/6 were enriched in cytokine–cytokine receptor interaction, antigen processing and presentation, natural killer cell-mediated cytotoxicity, Th1 and Th2 cell differentiation, etc. These results suggest that CDK4/6 plays an important role in the immunity of OC.

### The Correlation Between CDK4/6 Expression and Tumor-Infiltrating Immune Cells

**Figure 3:**
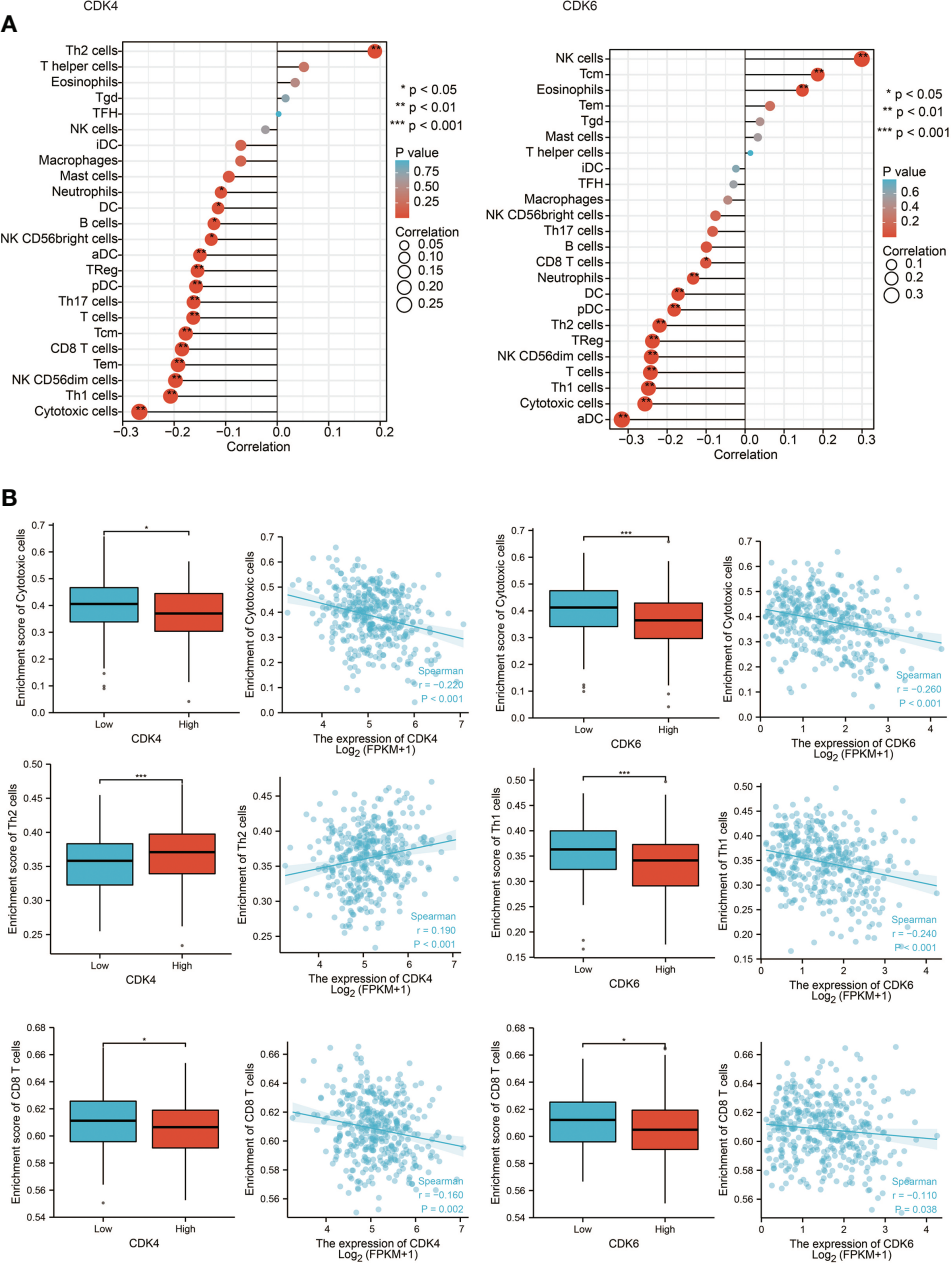
The correlation between CDK4/6 expression and tumor-infiltrating immune cells. **(A)** Correlation between the relative abundances of 24 immune cells and CDK4/6 expression level. The size of dots shows the absolute value of Spearman R. **(B)** Scatter plots and correlation diagrams showing the difference of cytotoxic cells, Th1/2 cells, and CD8^+^ T cells infiltration level between CDK4/6-high and CDK4/6-low groups.

Since the above results confirmed that CDK4/6 were negatively correlated with multiple immune molecules and pathways, we next focused on which immune cells and components affected CDK4/6. Therefore, we analyzed the Spearman correlation between the expression levels of CDK4/6 and the immune cell infiltration level quantified by single-sample gene set enrichment analysis (ssGSEA) in OC of the TCGA cohort. The abundance of most immune cells, such as cytotoxic cells, Th1 cells, CD8^+^ T cells, NK CD56 bright cells, etc., was negatively correlated with the expression levels of CDK4/6, with only a few positive correlations (**Figures 3A, B**, P <0.05). Consistently, we found that CDK4/6 expression levels were negatively correlated with the infiltration of most immune cells in the Tumor IMmune Estimation Resource (TIMER) database (**Figure S1B**). We also found a low infiltration level of various immune cells in samples with high copy numbers of CDK4/6 in OC (**Figure S1A**). On the other hand, we explored the relationship between chemokines, MHC molecules, immune activators, and the expression levels of CDK4/6 in the TISIDB database. The results indicated that CDK4/6 negatively regulated chemokines, MHC molecules, and immune activators in OC (**Figure S2**). In general, high expression of CDK4/6 might have a negative impact on the immune microenvironment, which inhibits the infiltration and activation of immune cells and reduces the antigen presentation ability of tumor cells.

### Prediction and Analysis of Upstream lncRNAs and miRNAs of CDK4/6

**Figure 4:**
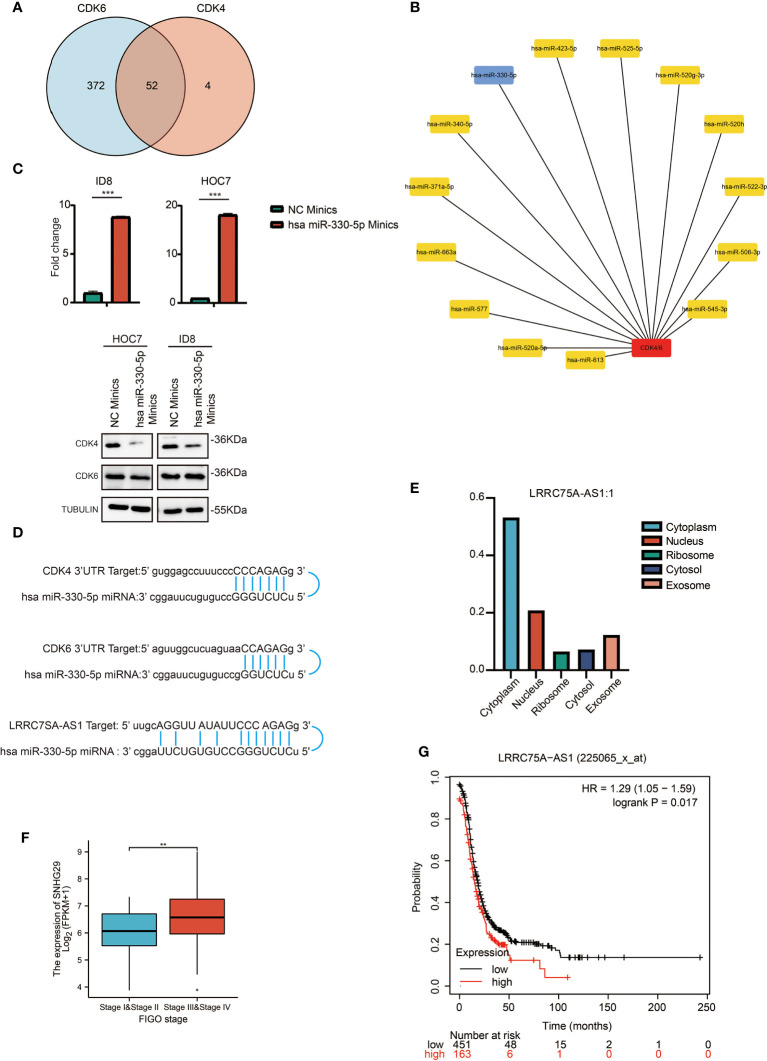
Prediction and analysis of upstream lncRNAs and miRNAs of CDK4/6. **(A)** Venn diagram showing the intersection of miRNAs targeting CDK4 and CDK6. **(B)** A significant miRNA-CDK4/6 regulatory network established by cytoHubba plug-in of cytoscape software. **(C)** QRT-PCR analysis of has miR-330-5p in ID8 and HOC7 treated with NC mimics and has miR-330-5p mimics for 72 h (upper). The p values were calculated using the Student t-test: ***P < 0.001. Western immunoblotting demonstrating expression level of CDK4/6 in ID8 and HOC7 treated with NC mimics and has miR-330-5p mimics for 72 h (lower). **(D)** Schematic diagram showing the regulatory relationship between ncRNAs and CDK4/6. **(E)** Bar graph showing the subcellular localization of LRRC75A-AS1. **(F)** Comparison of LRRC75A-AS1 expression between stages I & II and stages II & IV in the OC TCGA cohort. **p-value <0.01; ***p-value <0.001. **(G)** Kaplan–Meier analysis of the association between LRRC75A-AS1 expression and OS in OC. P values in **(F)** shows that there is a statistically significant difference in the expression level of LRRC75A (SNHG29) between the I/II and III/IV stages of ovarian cancer in the ovarian cancer TCGA database).

It has been generally recognized that ncRNAs participate in regulating gene expression. To determine which ncRNAs modulate CDK4/6, we first predicted potential upstream miRNAs that could bind to CDK4/6 and found 52 candidate miRNAs (**Figure 4A**). Then, we used the cytoHubba plug-in of Cytoscape software to establish a regulatory network of CDK4/6 and significantly associated miRNAs (**Figure 4B**). Finally, according to the regulatory relationships of miRNAs with the expression of target genes, we screened hsa-miR-330-5p as the only miRNA that was negatively correlated with CDK4 and CDK6 in OC *via* StarBase analysis (**Table 1**). We also found that the mimics of hsa-mir-330-5p upregulated CDK4/6 in ovarian cancer cell lines (**Figure 4C**). The same method was applied to identify lncRNAs upstream of hsa-miR-330-5p. Thirty lncRNAs were screened, and only LRRC75A-AS1 (SHNG29) was negatively correlated with hsa-miR-330-5p and positively correlated with CDK4/6 in OC according to the StarBase analysis (**Table 2**). As pictured in **Figure 4D**, hsa-miR-330-5p targeted CDK4/6 by binding with the 3’ untranslated region (UTR) of CDK4/6, and LRRC75A-AS1 paired with hsa-miR-330-5p in multiple bases. Subsequently, we explored the cytological location and clinical correlation of LRRC75A-AS1. The results indicated that LRRC75A-AS1 was mainly located in the cytoplasm and that higher LRRC75A-AS1 expression indicated a higher clinical stage (**Figures 4E, F**). Furthermore, the upregulation of LRRC75A-AS1 was positively linked to a poor prognosis in OC patients (**Figures 4G**). All these results suggested that LRRC75A-AS1 and hsa-miR-330-5p might be the best candidate regulatory ncRNAs of CDK4/6 in OC.

**Table 1:** Correlation analysis between has-mir-330-5p and CDK4/6 in OV determined by starBase database.

CDK4 AND CDK6	CDK4 R	P	CDK6 R	P
hsa-miR-15a-5p	−0.071	1.67E-01	−0.067	1.92E-01
hsa-miR-16-5p	−0.067	1.92E-01	−0.165	1.31E-03
hsa-miR-103a-3p	0.034	5.14E-01	0.084	1.05E-01
hsa-miR-107	0.035	4.97E-01	0.072	1.65E-01
hsa-miR-196a-5p	0.037	4.69E-01	−0.015	7.72E-01
hsa-miR-147a	0	1.00E+00	0	
hsa-miR-183-5p	0.165	1.36E-03	−0.014	7.80E-01
hsa-miR-216a-5p	0.044	3.96E-01	0.071	1.71E-01
hsa-miR-1-3p	−0.084	1.03E-01	0.069	1.84E-01
hsa-miR-15b-5p	−0.171	8.41E-04	−0.018	7.24E-01
hsa-miR-124-3p	−0.03	5.61E-01	0.012	8.16E-01
hsa-miR-142-5p	−0.067	1.94E-01	0.041	4.33E-01
hsa-miR-136-5p	−0.067	1.95E-01	0.037	4.77E-01
hsa-miR-149-5p	0.011	8.26E-01	0.171	8.62E-04
hsa-miR-185-5p	−0.15	3.58E-03	−0.067	1.98E-01
hsa-miR-195-5p	−0.168	1.09E-03	0.069	1.83E-01
hsa-miR-206	0.176	*0.000615	0.178	*0.00052
hsa-miR-34b-5p	−0.092	7.46E-02	0.042	4.11E-01
hsa-miR-326	−0.129	1.23E-02	−0.059	2.53E-01
hsa-miR-335-5p	−0.013	7.97E-01	0.126	1.45E-02
hsa-miR-196b-5p	0	0.00E+00	−0.039	4.53E-01
hsa-miR-424-5p	0.007	8.88E-01	0.03	5.57E-01
hsa-miR-448	−0.023	6.61E-01	−0.032	5.42E-01
hsa-miR-410-3p	−0.03	5.63E-01	0	0.00E+00
hsa-miR-486-5p	0.021	6.83E-01	−0.008	8.78E-01
hsa-miR-494-3p	−0.047	3.60E-01	0	0.00E+00
hsa-miR-497-5p	−0.196	1.28E-04	−0.033	5.21E-01
hsa-miR-512-3p	−0.016	7.62E-01	−0.048	3.50E-01
hsa-miR-520a-5p	−0.062	2.33E-01	−0.033	5.25E-01
hsa-miR-525-5p	−0.013	8.01E-01	−0.008	8.78E-01
hsa-miR-520g-3p	0.004	9.40E-01	−0.014	7.81E-01
hsa-miR-520h	−0.017	7.38E-01	0	0.00E+00
hsa-miR-522-3p	0.017	7.39E-01	0.026	6.11E-01
hsa-miR-506-3p	0.03	5.57E-01	0.113	2.89E-02
hsa-miR-545-3p	−0.041	4.23E-01	0.024	6.42E-01
hsa-miR-577	0.061	2.38E-01	−0.107	3.78E-02
hsa-miR-613	0	0.00E+00	0	0.00E+00
hsa-miR-663a	−0.018	7.32E-01	0	0.00E+00
hsa-miR-371a-5p	0.075	1.45E-01	0.089	8.34E-02
hsa-miR-340-5p	−0.027	6.05E-01	0.054	2.94E-01
hsa-miR-330-5p	−0.128	*0.0131	−0.113	*0.0291
hsa-miR-423-5p	−0.092	7.58E-02	0.033	5.18E-01
hsa-miR-874-3p	0.126	1.45E-02	−0.015	7.65E-01
hsa-miR-744-5p	−0.121	1.94E-02	0.09	8.18E-02
hsa-miR-885-5p	−0.109	*0.0344	−0.192	*0.000174
hsa-miR-216b-5p	0.135	8.69E-03	0.083	1.09E-01
hsa-miR-1252-5p	0	1.00E+00	0	1.00E+00
hsa-miR-1908-5p	0	0.00E+00	−0.015	7.67E-01
hsa-miR-449c-5p	0.037	4.69E-01	0.007	8.95E-01
hsa-miR-2682-5p	−0.059	2.51E-01	0.019	7.15E-01
hsa-miR-2467-3p	0.04	4.42E-01	0.104	4.37E-02
hsa-miR-6838-5p	0	9.93E-01	−0.005	9.18E-01

*These results are statistically significant. In the ovarian cancer TCGA database, the top 14 miRNAs calculated by the cytoscape software for correlation with CDK4/6 are shown in red.

**Table 2:** Correlation analysis between lncRNA and has-mir-330-5p or lncRNA and CDK4/6 in OV determined by starBase database.

lncRNA	miRNA	lncRNA and has-mir-330-5p R-value	lncRNA and has-mir-330-5p P-value	lncRNA and CDK4 R-value	lncRNA and CDK4 P-value	lncRNA and CDK6 R-value	lncRNA and CDK6 P-value
LRRC75A-AS1	hsa-miR-330-5p	−0.272	0.000000087*	0.206	0.0000525*	0.13	0.0114*
AC124312.3	hsa-miR-330-5p	−0.155	0.00252*	0.063	2.19E-01	0.243	1.70E-06
LINC00921	hsa-miR-330-5p	−0.1	0.0519*	−0.209	0.0000425*	0.094	6.64E-02
AC026471.1	hsa-miR-330-5p	−0.124	0.0163*	0.08	1.20E-01	0.069	1.79E-01
AC004943.2	hsa-miR-330-5p	−0.108	0.0366*	0.259	0.000000316*	0.11	0.033*
AC092127.1	hsa-miR-330-5p	0.05	3.34E-01	−0.093	7.05E-02	0.142	5.54E-03
AC138028.6	hsa-miR-330-5p	0.126	1.43E-02	−0.235	3.66E-06	0.127	1.33E-02
AC092384.3	hsa-miR-330-5p	0.049	3.41E-01	−0.066	1.98E-01	0.03	5.66E-01
AC113189.2	hsa-miR-330-5p	−0.018	7.27E-01	0.021	6.82E-01	0.135	8.45E-03
AC113189.4	hsa-miR-330-5p	0.043	4.11E-01	0.108	3.59E-02	0.149	3.73E-03
AC016876.2	hsa-miR-330-5p	0.097	6.01E-02	−0.123	1.68E-02	−0.121	1.85E-02
AC005304.2	hsa-miR-330-5p	0.009	8.55E-01	−0.033	5.17E-01	−0.022	6.71E-01
MEG3	hsa-miR-330-5p	0.031	5.47E-01	−0.124	1.54E-02	0.329	5.15E-11
SNHG14	hsa-miR-330-5p	−0.066	1.99E-01	−0.029	5.72E-01	0.195	1.37E-04
OIP5-AS1	hsa-miR-330-5p	−0.074	1.52E-01	−0.066	2.02E-01	0.141	5.87E-03
AC020891.2	hsa-miR-330-5p	−0.017	7.48E-01	0.084	1.02E-01	0.077	1.33E-01
AC090970.3	hsa-miR-330-5p	0.138	7.26E-03	0.063	2.24E-01	−0.015	7.65E-01
AC010999.3	hsa-miR-330-5p	0	0.00E+00	0	0.00E+00	0	0.00E+00
AC018904.2	hsa-miR-330-5p	0.065	2.07E-01	−0.144	4.94E-03	0.038	4.66E-01
NPTN-IT1	hsa-miR-330-5p	−0.004	9.38E-01	−0.222	1.24E-05	0.188	2.24E-04
AL031008.1	hsa-miR-330-5p	0.01	8.50E-01	−0.152	2.96E-03	−0.003	9.51E-01
SNHG9	hsa-miR-330-5p	−0.02	7.03E-01	−0.033	5.18E-01	−0.175	6.03E-04
AC012676.5	hsa-miR-330-5p	−0.008	8.71E-01	−0.096	6.33E-02	0.216	2.20E-05
SLX1A-SULT1A3	hsa-miR-330-5p	−0.06	2.48E-01	−0.054	2.91E-01	0.108	3.49E-02
AC007496.3	hsa-miR-330-5p	−0.043	4.11E-01	−0.104	4.25E-02	0.217	2.04E-05
AC026461.1	hsa-miR-330-5p	0	0.00E+00	0	1.00E+00	0	1.00E+00
AC040162.3	hsa-miR-330-5p	−0.011	8.29E-01	−0.111	3.11E-02	0.07	1.71E-01
AC126773.2	hsa-miR-330-5p	−0.036	4.90E-01	0.025	6.32E-01	0.358	6.49E-13
AC009022.1	hsa-miR-330-5p	0.066	1.99E-01	−0.177	5.56E-04	0.121	1.82E-02

*These results are statistically significant. In the ovarian cancer TCGA database, lncRNAs that are significantly related to hsa-mir-330-5p are shown in red.

### LRRC75A-AS1 and hsa-miR-330-5p Are Correlated With Immune Cell Infiltration in OC

**Figure 5:**
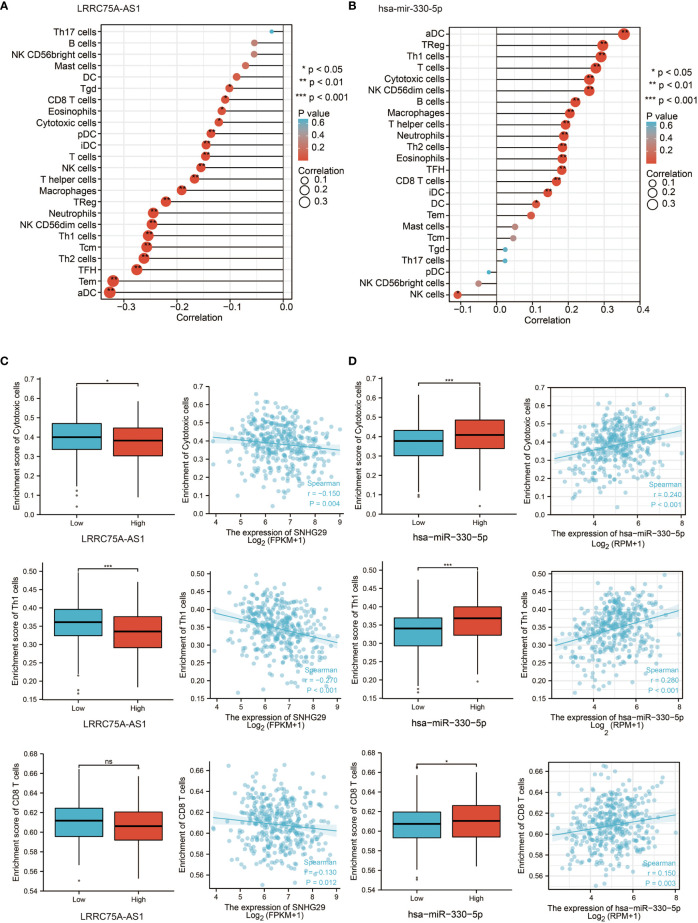
LRRC75A-AS1 and has-mir-330-5p are correlated with immune cell infiltration in OC. **(A)** Correlation between the relative abundances of 24 immune cells and LRRC75A-AS1 expression level in OC of the TCGA cohort. The size of dots shows the absolute value of Spearman R. **(B)** Correlation between the relative abundances of 24 immune cells and has-mir-330-5p expression level in OC of the TCGA cohort. The size of dots shows the absolute value of Spearman R. **(C)** Scatter plots and correlation diagrams showing the difference of cytotoxic cells, Th1/2 cells, and CD8^+^ T cells infiltration level between LRRC75A-AS1-high and LRRC75A-AS1-low groups. The p values were calculated using the Student t-test: ns as nonsense, *P < 0.05, **P < 0.01, ***P < 0.001, ****P < 0.0001. **(D)** Scatter plots and correlation diagrams showing the difference of cytotoxic cells, Th1/2 cells, and CD8^+^ T cells infiltration level between has-mir-330-5p-high and has-mir-330-5p-low groups. The p values were calculated using the Student t-test: ns as nonsense, *P < 0.05, **P < 0.01, ***P < 0.001, ****P < 0.0001.

According to previous results, CDK4/6 plays a negative role in immunity. Since LRRC75A-AS1 and hsa-miR-330-5p regulate CDK4/6 upstream, we focused on the impact of LRRC75A-AS1 and hsa-miR-330-5p on immunity. Interestingly, the effect of LRRC75A-AS1 on immunity was basically the same as that of CDK4/6 on immunity; in other words, LRRC75A-AS1 inhibited the infiltration of various immune cells (**Figure 5A**), while the influence of hsa-mir-330-5p on immunity was the opposite of that of CDK4/6; that is, hsa-mir-330-5p participated in immune activation (**Figure 5B**). This also confirmed the logical relationship in which LRRC75A-AS1 targeted hsa-miR-330-5p and then hsa-miR-330-5p inhibited CDK4/6. In addition, as depicted in **Figure 5C**, LRRC75A-AS1 expression was positively correlated with the infiltration levels of cytotoxic cells, Th1 cells, and CD8^+^ T cells, while hsa-miR-330-5p was positively correlated with cytotoxic cells, Th1 cells, and CD8^+^ T cells (P <0.05, **Figure 5D**). Therefore, the LRRC75A-AS1-has-mir-330-5p axis plays an important role in immunity by regulating CDK4/6 according to public data. Meanwhile, hsa-mir-330-5p mimics could upregulate the expression of ISGs and IFNs genes and activated immunity in ovarian cancer cell lines (**Figures S3A–D**), but further verification remains to be studied.

### CDK4/6 Inhibitor Palbociclib Exerts Antitumor Effects by Activating Immunity in OC Cell Lines

**Figure 6:**
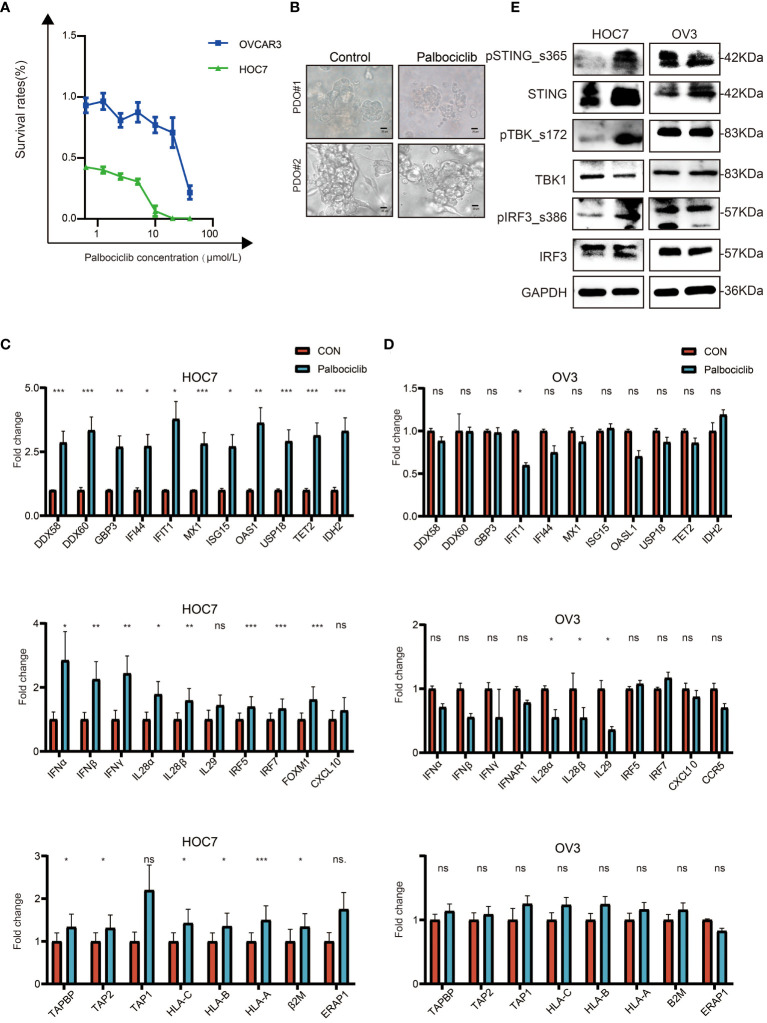
CDK4/6 inhibitor palbociclib exerts anti-tumor effect by activated immune in OC cell lines. **(A)** Cell viability in response to treatment with Palbociclib in OC cell lines. **(B)** Representative images of white light in PDO#1 and PDO#2 from OC were treated with Vehicle, 8 μM Palbociclib for 5 days, scale bars: 20 μm. **(C)** QRT-PCR analysis of ISG, IFN, and antigen-presented pathway in ID8 treated with Vehicle, 5 μM Palbociclib for 48 h. The p values were calculated using the Student t-test: ns as nonsense, *P < 0.05, **P < 0.01, ***P < 0.001. **(D)** QRT-PCR analysis of ISG, IFN, and antigen-presented pathway in OVCAR3 treated with Vehicle, 5 μM Palbociclib for 48 h. The p values were calculated using the Student t-test: ns as nonsense, *P < 0.05, **P < 0.01, ***P < 0.001. **(E)** Palbociclib-sensitive (ID8) and *de novo* Palbociclib-resistant (OVCAR3) OC cell lines were subjected to western blot analysis for STING pathway and GAPDH as a loading control.

OC cell lines showed different responses to the CDK4/6 inhibitor palbociclib. As shown in **Figure 6A**, HOC7 cells were more sensitive to palbociclib than OVCAR3 cells. Consistently, under the same conditions of palbociclib treatment, the sphere structure was severely damaged in some patient-derived organoids (PDOs), while still some OC PDOs were almost unaffected, which meant that OC PDOs also had different sensitivities to palbociclib (**Figure 6B**). We next sought mechanistic insight into the different responses after palbociclib treatment. qRT–PCR analysis of HOC7 and OVCAR3 revealed that palbociclib significantly upregulated the expression of ISGs, IFN-related genes, and antigen presentation genes in HOC7 (**Figure 6C**); however, palbociclib could not upregulate these genes in OVCAR3 (**Figure 6D**). Finally, we also found that under the same concentration of palbociclib, the STING pathway of HOC7 was upregulated and activated, while the STING pathway of OVCAR3 could not be activated (**Figure 6E**). These results suggested that ISG-, IFN-, and antigen presentation-related gene sets could be used to predict the response to palbociclib.

### The CDK4/6 Inhibitor Palbociclib Is at Least Partly Dependent on STING to Activate Immunity in OC

**Figure 7:**
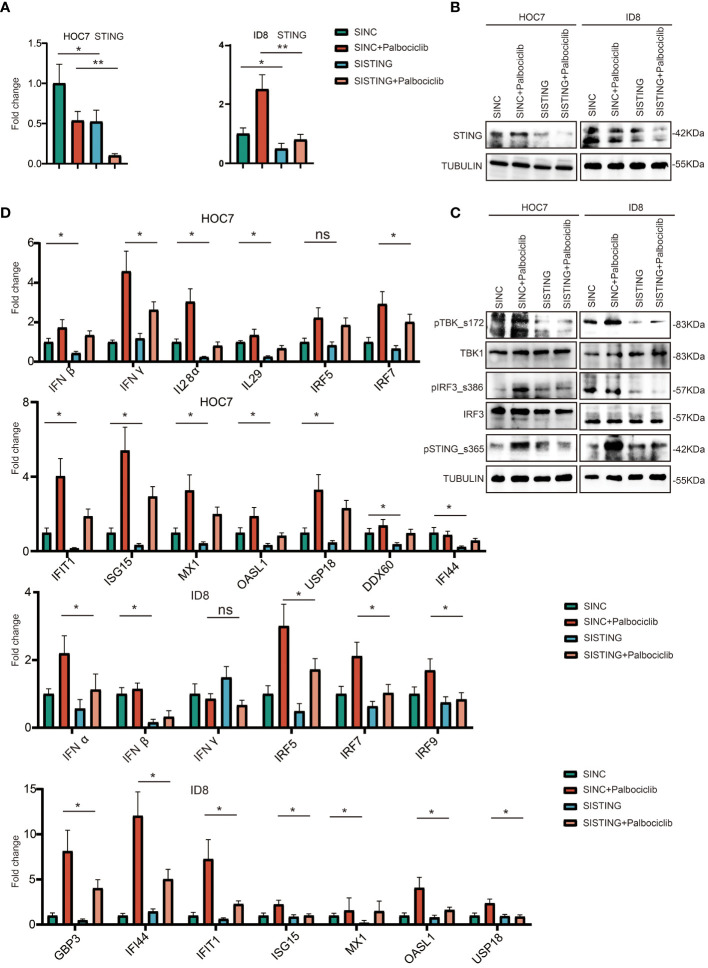
CDK4/6 inhibitor palbociclib is at least partly dependent on STING to activate immunity in OC. **(A)** QRT-PCR analysis of STING expression level in ID8 and HOC7 treated with siNC, siNC + Palbociclib, siSTING, and siSTING + Palbociclib for 72 h. The p values were calculated using the Student t-test: ns as nonsense, *P < 0.05, **P < 0.01, **(B)** Western immunoblotting demonstrating expression of STING in ID8 and HOC7 treated with siNC, siNC + Palbociclib, siSTING, and siSTING + Palbociclib for 72 h. Tubulin was used as a loading control. **(C)** Western immunoblotting demonstrating expression of STING pathway in ID8 and HOC7 treated with siNC, siNC + Palbociclib, siSTING, and siSTING + Palbociclib for 72 h. Tubulin was used as a loading control. **(D)** QRT-PCR analysis of ISG, IFN, antigen presentation related pathway in ID8 and HOC7 treated with siNC, siNC + Palbociclib, siSTING, and siSTING + Palbociclib for 72 h. The p values were calculated using the Student t-test: ns as nonsense, *P < 0.05, **P < 0.01.

To explore the potential mechanism of palbociclib-activated immunity, we knocked down STING expression in HOC7 cells using RNAi technology. As shown in **Figures 7A, B**, compared to the untreated sample, the STING mRNA and protein expression levels in the siSTING sample of HOC7 were decreased. We also found that siSTING partly restored the activation of the STING pathway by palbociclib (**Figure 7C**). Additionally, the increased expression levels of ISG-, IFN-, and antigen presentation-related genes were partly reversed by siSTING (**Figure 7D**). Hence, the immune response induced by palbociclib is at least partly dependent on STING.

### The CDK4/6 Inhibitor Palbociclib Suppresses Tumor Growth by Activating the Immune Microenvironment *In Vivo*

**Figure 8:**
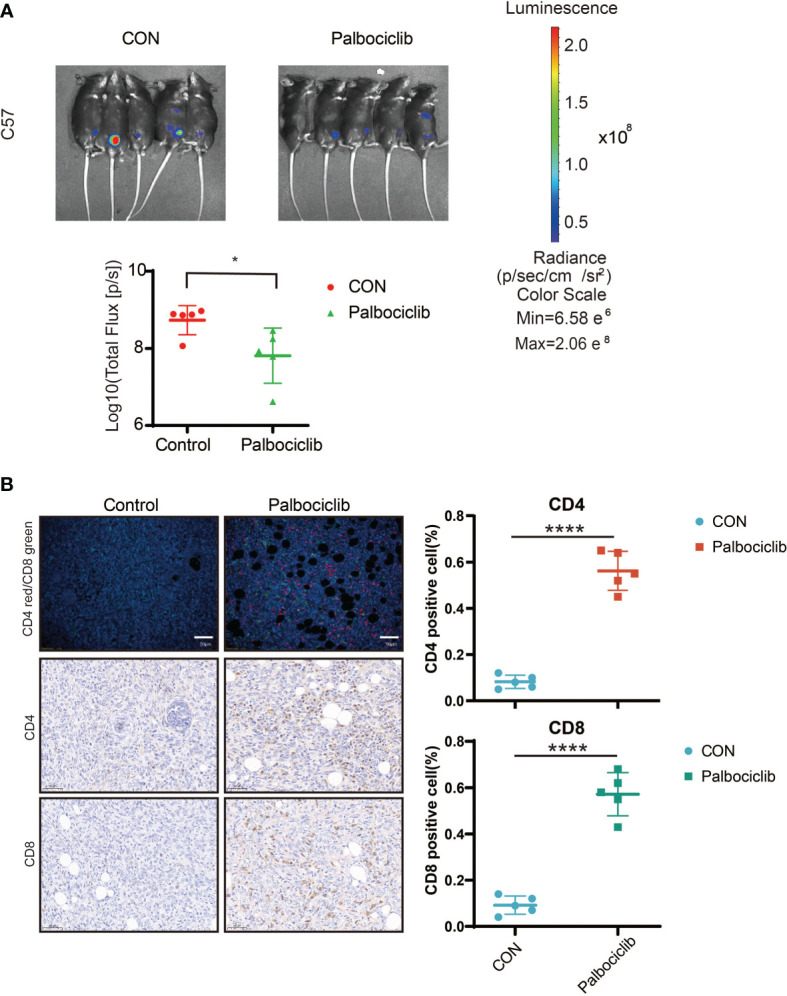
CDK4/6 inhibitor palbociclib suppresses tumor growth through activating immune microenvironment *in vivo.* ***(*A*)*** Luciferized ID8 xenograft models could reproducibly respond to the vehicle and Palbociclib could be serially followed by BLI imaging. Mice with established BLI signals were treated with 4 weekly treatments for vehicle (5% DMSO + 30% PEG300 + 5% Tween80 + ddH2O), Palbociclib (20 mg/kg, oral gavage, per day). All measurements are represented as Mean + SD. The p values were calculated using the Student t-test: *P < 0.05. **(B)** Representative images of IF/IHC with CD4/CD8 in Control and Palbociclib, red indicated CD4^+^ cells, green indicated CD8^+^ cells (Left), Scale bar, 20 μm. Positive cell (%) of indicated cells in right. Student’s t-test: ****P <0.0001.

Based on the impact of palbociclib on immunity *in vitro*, we explored the effect of palbociclib on the immune microenvironment *in vivo*. We used the ID8 abdominal cavity model of C57. Daily intragastric administration of palbociclib (40 mg/kg/d) delayed tumor growth much more potently than the control (**Figure 8A**). Additionally, we found that the levels of CD8^+^ and CD4^+^ T cells increased significantly in palbociclib-treated tumors, further suggesting a shift in the immune balance in favor of antitumor immunity (**Figure 8B**). Therefore, palbociclib suppresses tumor growth by activating the immune microenvironment *in vivo*.

## Discussion

Ovarian cancer is famous for its high fatality rate (14). Exploring the molecular mechanism of OC is conducive to finding effective therapeutic targets (15, 16). Increasing evidence has suggested that CDK4/6 play important roles in the progression and prognosis of multiple cancers, including OC (17). However, knowledge about CDK4/6 in OC remains inadequate and needs to be further explored.

First, we find that CDK4/6 are overexpressed in a variety of tumor tissues, including OC, and that high expression of CDK4/6 is associated with a poor prognosis in OC patients. Dai et al. propose that CDK4 regulates prometastatic inflammatory cytokine signaling, whereas CDK6 mainly controls DNA replication and repair processes, indicating that CDK4 and CDK6 facilitate tumor growth and progression in multiple cancers (18).

Second, inhibition of CDK4/6 could activate multiple immune components, especially CD4/CD8^+^ cells. Mechanistically, a CDK4/6 inhibitor enhanced the secretion of IFN by activating the STING pathway and thus enhanced tumor antigen presentation ability. To date, many studies have suggested that CDK4/6 are cyclin-dependent kinases activated in response to proliferative signaling but also have significant effects on cancer cells and the tumor microenvironment (19–21). Charles et al. found that CDK4/6 inhibitor treatment led to the production of drug-induced HLA ligands from the G1/S phase transition, which have the highest chance of being recognized by T cells (22). Lelliott *et al.* demonstrated a novel action of CDK4/6 inhibitors in promoting the phenotypic and functional acquisition of immunological T cell memory (23). In terms of the mechanism of CDK4/6 in regulating immunity, in 2017, a report revealed that CDK4/6 inhibitors increase the intracellular levels of double-stranded RNA (dsRNA). This in turn stimulates the production of type III IFNs and hence enhances tumor antigen presentation (12). In our study, we found that dsRNA played an important role in immune activation by CDK4/6 inhibitors and that the STING pathway was also responsible for this effect. The reason might be that CDK4/6 inhibitors could also cause the accumulation of double-stranded DNA (dsDNA) by inducing DNA damage (24, 25). Additionally, under the same concentration of CDK4/6 inhibitor treatment, ISG, IFN, and antigen presentation-related genes were upregulated in the relatively sensitive cell line HOC7, while these genes did not change significantly in the relatively drug-resistant OVCAR3, indicating that a signature consisting of ISGs, IFNs, antigen presentation-related genes could be used to predict the response to CDK4/6 inhibitors (26).

Third, we also found that the LRRC75A-AS1-has-mir-330-5p axis was upstream of CDK4/6 and could inhibit the immunity of OC patients by upregulating CDK4/6. As reported, ncRNAs play crucial roles in the development of tumors (27, 28). However, the role of LRRC75A-AS1-has-mir-330-5p in ovarian tumors is still unknown. In our study, to identify the upstream regulatory miRNAs of CDK4/6, we applied PITA, RNA22, miRmap, microT, miRanda, PicTar, and TargetScan to predict possible miRNAs that target CDK4/6. Fifteen miRNAs were ultimately obtained. Only hsa-miR-330-5p and CDK4/6 were negatively correlated in OC. We also explored the negative impact of hsa-miR-330-5p on immunity in OC from the TCGA cohort. Additionally, Fu et al. reported that the expression of miR-330-5p was downregulated in OC cell lines and samples compared to normal controls (29). Sun et al. found that miR-330-5p levels increased but Tim-3 levels decreased, leading to M1 polarization and insulin tolerance in diabetic mice (30). Moreover, we used the Cytoscape plug-in to identify the top 30 CDK4/6-associated and hsa-mir-330-5p-related lncRNAs, of which LRRC75A-AS1 and AC004943.2 negatively regulated hsa-mir-330-5p and positively regulated CDK4/6 in OC (**Table 2**). We finally determined the LRRC75A-AS1-hsa-miR-330-5p axis through the impact of LRRC75A-AS1 on survival and tumor staging. Additionally, we found that LRRC75A-AS1 positively regulated immunity in OC *via* analysis of the TCGA cohort. A previous study revealed the relationship between LRRC75A-AS1 and hsa-mir-330-5p (31), but few reports have addressed the impact of LRRC75A-AS1 on immunity, which needs further study (32).

In summary, we found that CDK4/6 were highly expressed in multiple types of human cancers (including OC) and negatively correlated with a good prognosis in OC patients. Then, according to public data, we identified the LRRC75A-AS1-hsa-miR-330-5p axis as the upstream regulatory mechanism of CDK4/6 in OC. Furthermore, our study suggested that the LRRC75A-AS1-hsa-miR-330-5p-CDK4/6 axis might exert its antitumor function by inhibiting the antigen presentation ability of tumor cells and by inhibiting immune cell infiltration and activation. However, these results need to be validated by basic experiments. Finally, we verified that CDK4/6-mediated activation of immunity at least partly depends on the STING pathway in OC cell lines and animal models. A signature, namely, ISGs, IFN-related genes, and antigen presentation-related genes might be used as biomarker to predict the response to CDK4/6 inhibitors.

## Data Availability Statement

The datasets presented in this study can be found in online repositories. The names of the repository/repositories and accession number(s) can be found in the article/**Supplementary Material**.

## Ethics Statement

The studies involving human participants were reviewed and approved by S360. The patients/participants provided their written informed consent to participate in this study. The animal study was reviewed and approved by TJH-202007001.

## Author Contributions

CS: Conceptualization and Funding acquisition. CL: Investigation, Project administration, and Methodology. YH: original draft, Data curation, Software, and Formal analysis. LY, TQ, FL, XiL, YL, DH, RX, XQ, EG, BY, JF, XioL, YF, SL, ZW, YD, WW, WL, XY, JL, WP, LZ, YC, and JZ: Supervision and Validation. All authors contributed to the article and approved the submitted version.

## Funding

This study is supported by the Fundamental Research Funds for the Central Universities, Hust:2020JYCXJJ016 and 2020JYCXJJ014.

## Conflict of Interest

The authors declare that the research was conducted in the absence of any commercial or financial relationships that could be construed as a potential conflict of interest.

## Publisher’s Note

All claims expressed in this article are solely those of the authors and do not necessarily represent those of their affiliated organizations, or those of the publisher, the editors and the reviewers. Any product that may be evaluated in this article, or claim that may be made by its manufacturer, is not guaranteed or endorsed by the publisher.
