# Supplementary material for: The Immunological Role of CDK4/6 and Potential Mechanism Exploration in Ovarian Cancer
Source: Front Immunol. 2022 Jan 14;12:799171. doi: 10.3389/fimmu.2021.799171 (PMC8795791; doi:10.3389/fimmu.2021.799171)

**The immunological role of CDK4/6 and potential mechanism exploration in ovarian cancer**

Chen Liu^1#^, Yuhan Huang^1,2#^, Yaoyuan Cui^1^, Jun Zhou^3^, Xu Qin^1^, Li Zhang^1^, Yuan Li^1^, Ensong Guo^1^, Bin Yang^1^, Xi Li^1^, Junpeng Fan^1^, Xiong Li^1^, Yu Fu^1^, Si Liu^1^, Dianxing Hu^1^, Rourou Xiao^1^, Zhuozi Wang^1^, Yingyu Dou^1^, Wei Wang^1^, Wenting Li^1^, Xiaohang Yang^1^, Jingbo Liu^1^, Wenju Peng^1^, Tianyu Qin^1^, Lixin You^1^, Funian Lu^1^, Chaoyang Sun^1*^

^1^ Department of Obstetrics and Gynecology, Tongji Hospital, Tongji Medical College, Huazhong University of Science and Technology, Wuhan 430030, China

^2^ Department of Obstetrics and Gynecology, Shanghai General Hospital, Shanghai Jiao Tong University School of Medicine, Shanghai, China

^3^ Department of pediatrics, Tongji Hospital, Tongji Medical College, Huazhong University of Science and Technology, Wuhan 430030, China

**Correspondence to*: suncydoctor@gmail.com

^#^These authors have contributed equally to this work.

**FigureS1. The relationship of immune cell infiltration with CDK4/6 level in OC**

1. The infiltration level of various immune cells under different copy numbers of CDK4/6 in OC
2. The correlation of CDK4/6 expression level with B cell, CD8^+^ T cell, CD4^+^ T cell, macrophage, neutrophil, or dendritic cell infiltration level in OC.

**FigureS2. The co-expression of CDK4/6 and immune-related genes**

1. Heat map showing the relationship between CDK4/6 expression level and chemokines, MHC molecules, immune activators in pan-cancer on TISIDB database.

**FigureS3. The immunological role of** **has miR-330-5p in ovarian cancer**

1. QRT-PCR analysis of ISG, IFN, and antigen-presented pathway in HOC7 treated with NC minics and has miR-330-5p minics for 72h.
2. QRT-PCR analysis of ISG, IFN, and antigen-presented pathway in ID8 treated with NC minics and has miR-330-5p minics for 72h.
3. Western immunoblotting demonstrating expression of STING pathway in HOC7 treated with NC minics and has miR-330-5p minics for 72h.
4. Western immunoblotting demonstrating expression of STING pathway in ID8 treated with NC minics and has miR-330-5p minics for 72h.
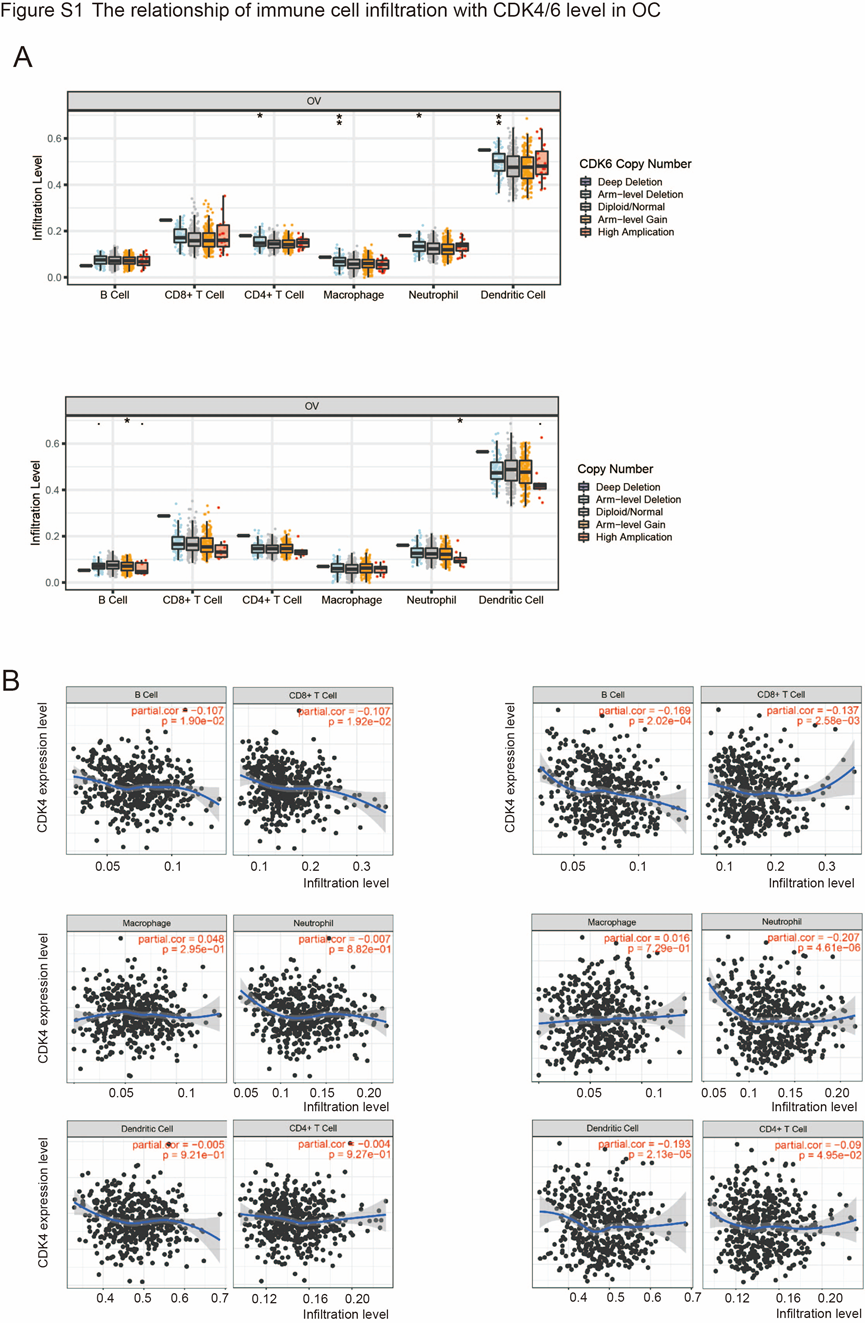


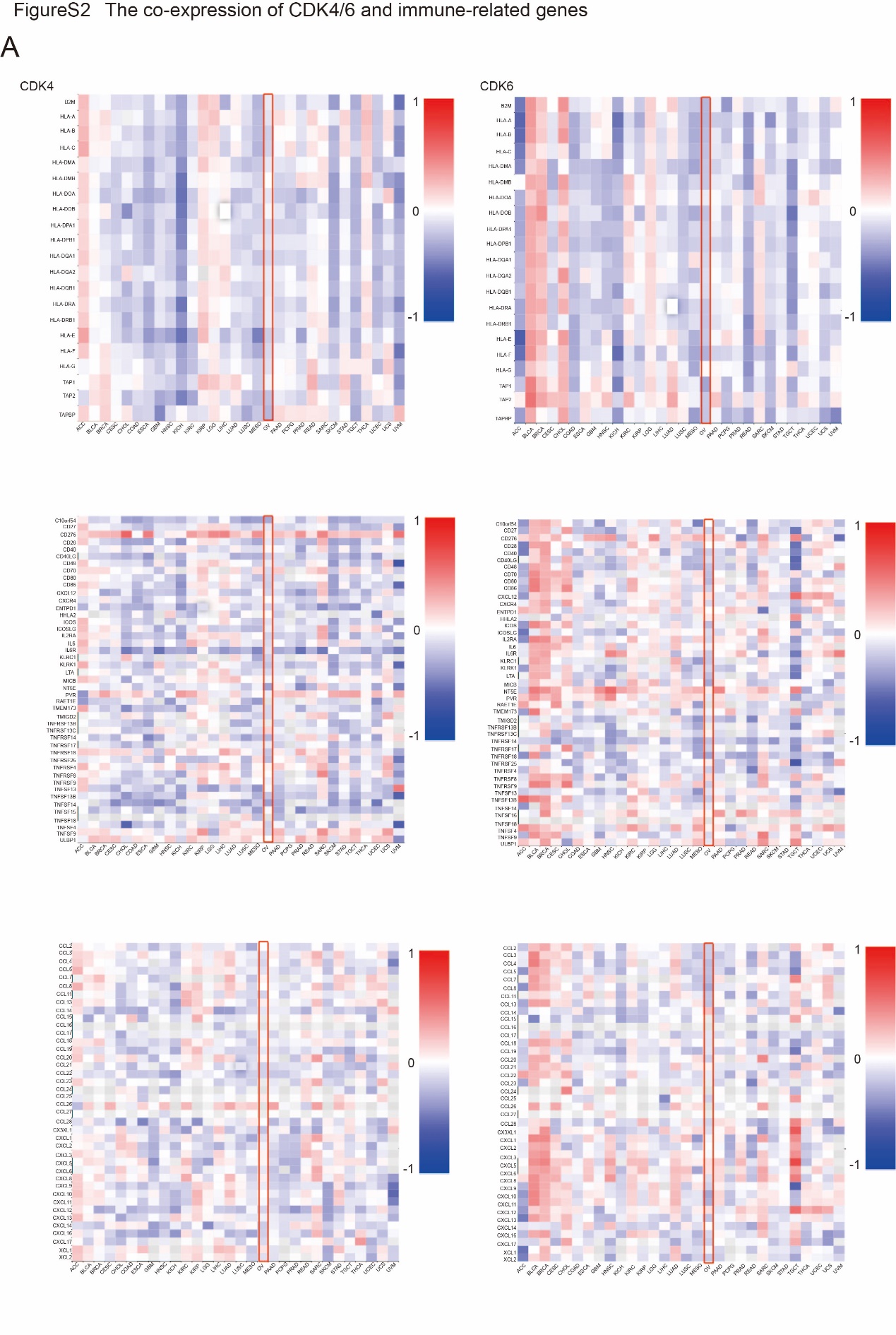


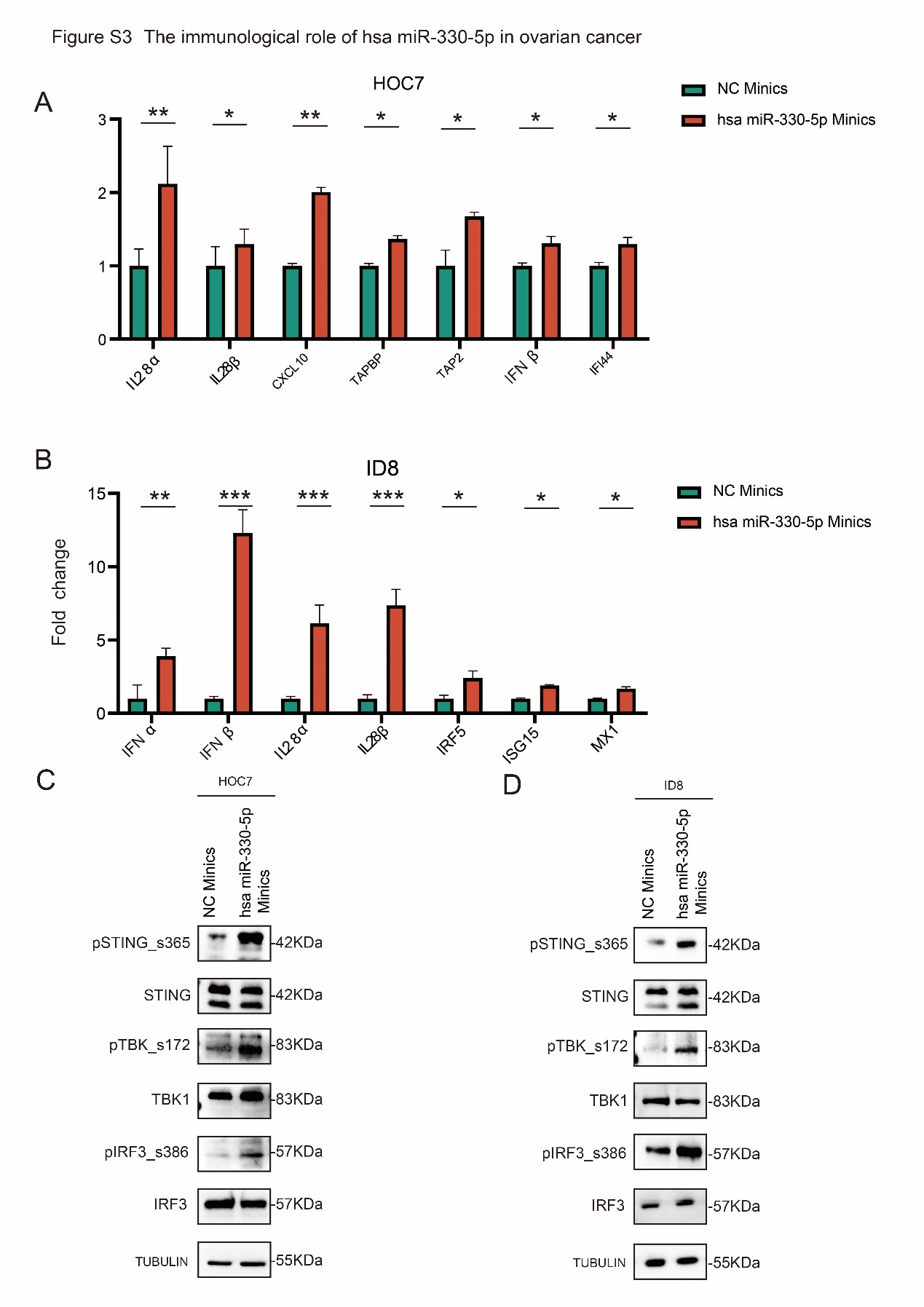

Supplement: Supplementary file 1 [file DataSheet_1.docx]
